# *Helicobacter pylori* increases the risk of carotid plaque formation: a clinical evidence

**DOI:** 10.1080/07853890.2021.1927169

**Published:** 2021-08-25

**Authors:** Haiqing Liang, Shuzhu Lin, Yongjian Ji, Yang Xiao, Guifang Zheng

**Affiliations:** a Department of Internal Medicine, Dezhou People's Hospital, Dezhou, China; b Department of Clinical Laboratory, Dezhou People's Hospital, Dezhou, China

**Keywords:** *Helicobacter pylori*, lesion formation, cervical vessel, dyslipidemia, BMI

## Abstract

**Background and aim:** Since the relation between *Helicobacter pylori* (*H. pylori*) and atherosclerosis has been evidenced, we aimed to analyze whether there is a relationship between the patient's *H. pylori* infection and age, gender, BMI, blood lipids, and carotid plaque formation.

**Methods:** 810 patients from January 2016 to December 2019 were enrolled in this study, and divided the subjects into *H. pylori* (+) group and *H. pylori* (-) group based on the results of UBT. To analyze whether *H. pylori* infection is related to gender, age, BMI, blood lipids, and neck vascular plaque formation.

**Results:** The single-factor analysis showed that the BMI ≥ 25kg/m^2^, triglycerides >1.7 mmol/l, the formation of cervical plaques were significantly higher in patients infected with *H. pylori* in compared to normal cases. Also, multi-variant logistic regression analysis showed that *H. pylori* infection affects the BMI ≥ 25kg/m^2^ and triglycerides >1.7 mmol/l to induce vascular plaque. Also, we showed that patients with *H. pylori* infection are 1.424 times higher than the non-infected group to have triglycerides more elevated than 1.7mmol/l.

**Conclusion:** In this study, we conclude that *H. pylori* infection is an independent risk factor for higher BMI (>25), triglyceride (>1.7 mmol/l), and neck vascular plaque formation. The multi-variant analysis showed that patients with *H. pylori* infection are prone to have higher BMI, triglycerides, and neck vascular plaque formation over 1.4-times higher in non-infected individuals.

**KEY MESSAGES**
- *H. pylori* infection is an independent risk factor for higher BMI, triglyceride, and neck vascular plaque formation.
- *H. pylori* can accelerate vascular plaque formation through increasing BMI and triglyceride.

## 1. Introduction

*Helicobacter pylori* (*H. pylori*), a Gram-negative bacillus bacterium, has a high prevalence ofover50% worldwide, especially in developing countries, with a0.5%–1% increase per year [1]. Recent studies have pointed out that *H. pylori* infection, as a chronic infection, is not only related to gastrointestinal diseases but also related to a variety of parenteral diseasesespecially gastrointestinal cancer [2,3]. It has been suggested that approximately 10% of *H. pylori* infectionsdevelop peptic ulcer disease, 1 to 3% gastric adenocarcinoma, and less than 0.1% gastric mucosa-associated lymphoid tissue (MALT) lymphoma [4,5]. *H. pylori* is considered as a type I carcinogen (for the International Agency for Research on Cancer), and gastric cancer is the fifth most common malignancy worldwide [5].In addition, previous studies have shown that lipid metabolism and the occurrence/progression of atherosclerosis are closely related to chronic infections [6].

Hyperlipidaemia, as a clear independent risk factor for cardiovascular and cerebrovascular diseases, has a non-negligible relationship with the occurrence and development of many conditions such as diet structure, obesity, exercise, genetics, andinflammation. Previous publications have confirmed that changes in lipid metabolism can lead toan incrementin the growth of various bacteriaand the imbalanced intestinal flora can aggravate the abnormal lipid metabolism [4,6].

Cervical vascular plaque formation is considered to be evidence of carotid atherosclerosis. Atherosclerosis (AS) is initially caused by abnormal lipid metabolism in the body. It develops from the arterial intima and it may progress to block the vascular lumen and induce ischemiaof corresponding tissue [7]. In this study, we evaluate carotid atherosclerosis because it is a relatively easy-to-detect blood vessel by B-ultrasound measurement. Some experimental studies have shown that *H. pylori*can affectthe normal flora of the intestine [8]. This processis considered as a part of the cause of cardiovascular and cerebrovascular diseases in *H. pylori*-infected cases [9–11]. Relevant literature points out that *H. pylori* infection will promote atherosclerosis's occurrence and development, and the mechanism is considered to be achieved through inflammation, oxidative stress, and immune responses. The stability of atherosclerotic plaque is a critical issue in assessing the severity of cardiovascular and cerebrovascular diseases. Homocysteine (Hcy), an intermediate product of thiosine metabolism, is an important factor in atherosclerotic plaque stability. Relevant studies have shown that an increased level of Hcy could induce a significant effect on the occurrence and development of atherosclerosis and thromboembolic diseases [12,13]. Infection can cause chronic gastritis and peptic ulcers, and other gastrointestinal diseases, which may affect the absorption and utilisation of vitamins, causing Hcy to accumulate in the body. The accumulated Hcy will worsen atherosclerosis, which is mediated by immunity [14]. In this study, we aimed to analyse whether there is a relationship between the patient’s *H. pylori* infection and age, gender, BMI, blood lipids, and carotid plaque formation.

## 2. Patients and methods

### 2.1. Study design

**Figure 1.**
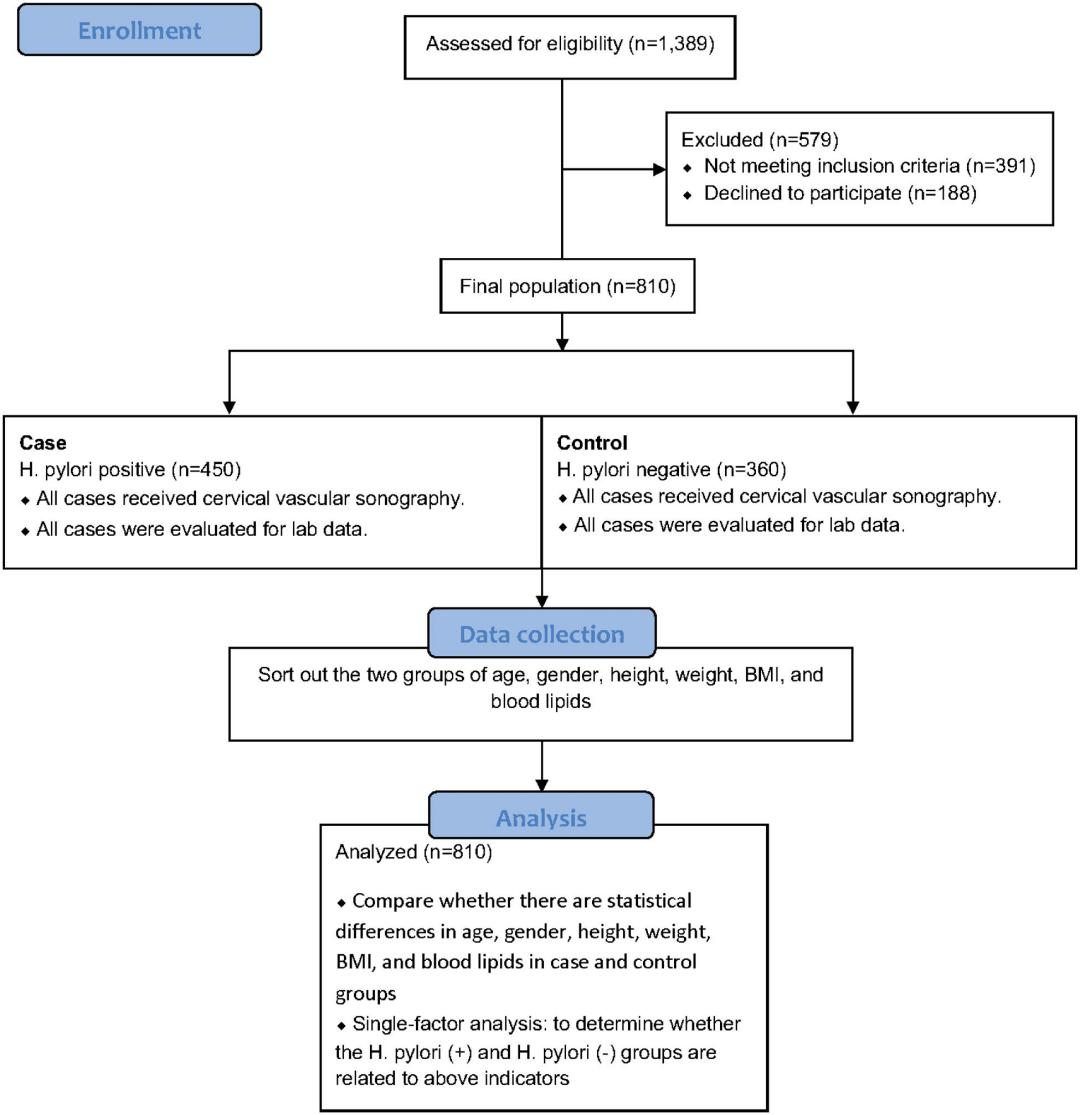
Road map of the study.

In this cross-sectional investigation, of 1,389 individuals, we consecutivelycollected data of 810 patients who were treated at Dezhou People's Hospital from January 2016 to December 2019, based on inclusion criteria. This study was accomplished according to the guidelines of the Helsinki Declaration and verified by the Clinical Research Ethics Committee of the Dezhou People's Hospital, Dezhou, Shandong, China. All participants signed written informed consent before the study. The patients were included based on the following criteria: complete anthropometricinformation, a positive ^13^C-urea breath test (^13^C-UBT) results, blood biochemical results, and cervical vascular ultrasound data. The patients who had a history of hyperlipidaemia and/or chronic disorders, such as coronary heart disease, stroke, hypertension, and diabetes as wells as patients with incomplete clinical data were excluded from the study. The research map was illustrated in Figure 1.

### 2.2. Detection of H. pylori infection

To diagnose patients with *H. pylori* infection, a two-step fasting ^13^C-UBT test was carried out. The ^13^C-UBT was performed under the manufacturer’s protocol: In brief, participants come in for a 6–8 hs fast, and a baseline breath specimen was collected. After 10 min, participants drank 100 mL of water containing 75 mg of ^13^C isotope-labeled urea (Beijing Boran Pharmaceutical Co. Ltd., China). 30 min later, a second sample of respiration was gathered and then analysed by infra-red heterodyne ratiometry (Beijing HuahengAnbang Company, China). Based on the cut-off delta-over-baseline (DOB) values, subjects were classified into the *H. pylori*-negative group and the *H. pylori*-positive group [15]. To determine the DOB, a receiver-operating characteristic curve analysis was done. DOB less than 4 was referred to as a negative infection, and DOB ≥4 as a positive infection.

### 2.3. Anthropometric assessments

Demographic data such as age, gender, weight, height, body mass index (BMI), blood pressure, personal medical history, and current medication use were collected for the study as explained [16]. The weight measurement was justified to 0.1 kg, and the height was justified to 0.1 cm. The BMI was determined by calculating the weight (kg)/the square of height (m^2^). Systolic blood pressure (SBP) and diastolic blood pressure (DBP) were evaluated on the right arm using an electronic sphygmomanometer, and three measured values of each participant were noted, and the mean values were recorded as the final data. The participant was determined to be hypertensive once the DBP > 80 mmHg or the SBP was > 130 mmHg or on anti-hypertensive drugs.

### 2.4. Measuring atherogenic plasma lipids

Plasma concentrations of traditional atherogenic lipid particles, including triglycerides (TG), total cholesterol (TC), high-density lipoprotein (HDL), Low-density lipoprotein (LDL)were assayed using the Biosystems kits (Biosystems S.A., Barcelona, Spain).

### 2.5. Ultrasonography of carotid artery and atherosclerosis lesion detection

To determine the presence of the atherosclerosis lesions, the distance between the aortic intima and the media-adventitia interface, defined as carotid intima-media thickness (CIMT), was measured in cervical vessels using the Philips iE33 colour Doppler ultrasonographer (Royal Dutch Philips Electronics Ltd., Amsterdam, Netherlands) utilising an 8.5-MHz linear probe. The participants underwent to the occiput and supine, fully exposed the chest and neck, and the common carotid artery was checked by placing the probe in a visible position. The two-dimensional ultrasound images of each common carotid artery, displaying the anterior and posterior walls of the vessel, were used to carry out all procedures. The CIMT was measured at the 10 mm proximal to the artery bifurcation of the left and right common carotid arteries. The mean CIMT value of the three independent measurements obtained from each side was calculated for each participant. The carotid atherosclerotic lesion was defined as the CIMT more than 1.4 mm or the detection of focal wall thickening at least 50% higher than that of the surrounding artery wall as explained [17].

### 2.6. Statistical methods

The data were described by the frequency percentage, and the comparison between the two groups was tested by κ^2^ test. First, a single-factor analysis was performed to screen statistically significant indicators as independent variables, and then multi-variantlogistic regression analysis was performed to find indicators independently related to infection. All statistical analysis was implemented in SPSS 25.0, *p* values < .05 were considered statistically significant.

## 3. Results

Table 1 represents the detailed statistical analysis and subject characteristics in *H. pylori*-positive and negative groups. Of the 810 participants who met the inclusion criteria in the study, 450 members were detected in the *H. pylori* infection and 360 members(44.4%) were in the *H. pylori*-negative. *H. pylori*-negative cases were considered as the control group. We observed cervical vascular plaque formation in 291participants(35.9%), and 519 participants (64.1%)had no cervical vascular plaque formation. The carotid atherosclerosis was detected in 186(51.7%) *H. pylori*-positive and 105 (20.2%) *H. pylori*-negative participants. *H. pylori*-positive participants suffered a significantly higher incidence of carotid atherosclerosis (41.3% vs. 29.2%, *p* = .006) and thicker CIMT (0.69 ± 0.09 vs. 0.77 ± 0.01, *p* = .001) compared with *H. pylori*-negative subjects. Among participants, 445 and 365 cases had BMI < 25 kg/m^2^ and ≥25 kg/m^2^, respectively. The percentage of cases with BMI ≥25 kg/m^2^ was significantly higher in the *H. pylori*-positive group than *H. pylori*-negative group. In *H. pylori*-positive group, a significantly higher percentage (50.40%) of cases had BMI ≥25 kg/m^2^. There were 521 and 289 participants with TG ≤ 1.7 mM and >1.7 mM, respectively, and the rate of *H. pylori* infection in cases with TG >1.7 mM (64.80%) was significantly higher than those with triglyceride ≤ 1.7 mM (35.20%). The frequency percentage of cases with TG >1.7 mM was significantly higher in *H. pylori*-positive group than *H. pylori*-negative group. The results of the univariate analysis showed that the BMI of ≥25 kg/m^2^, triglycerides higher than 1.7 mmol/l, and the presence of cervical vascular lesion were significantly higher in patients with *H. pylori* infection. However, gender, age, TC, LDL, and HDL showed no statistically significant difference between the two groups (Table 1). Multivariate non-conditional logistic regression analysis was performed on the indicators related to bacterial infection (Table 2), and it was concluded that *H. pylori* infection is independent risk factor for BMI ≥25 kg/m^2^ (*p* = .020; OR = 1.426 95% CI: 1.058–1.922), triglycerides >1.7 mM (*p* = .026; OR = 1.424 95% CI: 1.042–1.944, vascular lesion formation (*p* = .027; OR = 1.467 95% CI: 1.045–2.060).

**Table 1.** The comparison of *H. pylori* infection rate among various research indicators.

	Hp infection			
	Negative	Positive		
Index grouping	No. (%)	No. (%)	X2 value	p Value
Gender			0.194	0.659
Male	188 (52.20%)	242 (53.80%)		
Female	172 (47.80%)	208 (46.20%)		
Age			4.191	0.123
<44	151 (41.90%)	172 (38.20%)		
45 − 55	173 (48.10%)	212 (47.10%)		
>55	36 (10.00%)	66 (14.70%)		
Cervical vessel plaque			12.861	<0.001
No	255 (70.80%)	264 (58.70%)		
Yes	105 (29.20%)	186 (41.30%)		
BMI			11.850	0.001
<25	222 (61.70%)	223 (49.60%)		
≥25	138 (38.30%)	227 (50.40%)		
Triglycerides			5.892	0.015
<1.7	248 (68.90%)	273 (60.70%)		
>1.7	112 (31.10%)	177 (39.30%)		
Total cholesterol			0.409	0.523
<5.72	328 (91.10%)	404 (89.80%)		
>5.72	32 (8.90%)	46 (10.20%)		
High density lipoprotein			1.848	0.174
<1.55	278 (77.20%)	365 (81.10%)		
>1.55	82 (22.80%)	85 (18.90%)		
Low density lipoprotein			1.446	0.229
<3.1	234 (65.00%)	274 (60.90%)		
>3.1	126 (35.00%)	176 (39.10%)		

**Table 2.** Logistic regression analysis of factors affected by HP infection.

Index*	B	S.E.	Wald	df	p	OR (95%CI)
Plaque formation	0.383	0.173	4.890	1	.027	1.467 (1.045, 2.060)
BMI	0.355	0.152	5.415	1	.020	1.426 (1.058, 1.922)
Triglycerides	0.353	0.159	4.935	1	.026	1.424 (1.042, 1.944)
Constant	−1.763	0.257	47.017	1	.000	0.171 (−)

*National assignment: Cervical blood vessel formation plaque Assignment: None = 0, Yes = 1; BMI < 25 and >25 are assigned values of 0 and 1, respectively; triglycerides ≤1.7 mmol/l and >1.7 mmol/l are assigned values of 0 and 1; the presence or absence of Helicobacter pylori infection were assigned respectively: none = 0, with = 1.

## 4. Discussion

In the current study, the single-factor analysis showed that the rate of BMI ≥25 kg/m^2^, TG >1.7 mM, and the formation of cervical lesions were significantly higher in *H. pylori*positive subjects in comparison to normal cases. Importantly, multivariant logistic regression analysis showed that *H. pylori* infection is an independent risk factor for high levels of TG and carotid atherosclerosis in Chinese subjects.

Abnormal lipid profile, such as high plasma levels of TG, are the main risk factors of atherosclerosis. *H. pylori* infection is an independent risk factor for dyslipidemia [10,12,13,18,19]. In the present study, there was no significant difference in plasma levels of TC, LDL-C, and HDL-C between *H. pylori*-positive and negative participants. However, we found that the rate of TG levels >1.7 mM in *H. pylori*-positive group is 1.4 times higher than that in *H. pylori*-negative group. In line with our study, other investigations showed the relationship between *H. pylori* infection and elevated plasma levels of TG [18–20]. *H. pylori* infection has been found to affect lipid metabolism by various mechanisms. *H. pylori*infection can cause long-term, chronic inflammatory by inducing expression of various inflammatory cytokines such as tumour necrosis factor-α (TNF- α), which suppresses lipoprotein lipase, leading to the trafficking of lipids from tissues and increased plasma levels of TG [21]. Moreover, *H. pylori* infection can cause changes in intestinal microbes and bacterial environment, significantly reduce the probiotics in the intestinal flora, and lead to abnormal lipid metabolism [22–24].

The relationship between *H. pylori* infection and atherosclerosis has been inconsistent and sometimes controversial in various cross-sectional investigations. A positive relationship between *H. pylori* infection and coronary artery diseases (CAD) has been reported by epidemiologic studies that showed the higher prevalence of serologically verified *H. pylori* infection in patients with angiographically confirmed CAD [25–27]. Carotid atherosclerosis is an objective indicator of atherosclerosis, which shows a significant causative relationship with cardiovascular events. Of note, a positive association has been found between *H. pylori* infection and carotid atherosclerosis with elevated CIMT [28–32]. In contrast, a meta-analysis of 18 epidemiological studies including 10,000 patients indicated no positive association between *H. pylori* infection and CAD [33]. Differences in the study protocol (qualitative and quantitative analysis) and imaging modalities applied for diagnosis of CAD (coronary angiogram) and carotid atherosclerosis (carotid ultrasound) can interpret, at least partially, the significant difference inconsistency on the association between *H. pylori* infection and CAD versus carotid atherosclerosis. Carotid ultrasound could easily detect early atherosclerotic lesions, while coronary angiogram could not. The main character of atherosclerosis is an increase in the arterial CIMT that cannot be detected with an angiogram, while carotid ultrasonography is a sensitive and ideal non-invasive imaging approach to diagnose and monitor the atherosclerosis lesion progression [34]. However, the carotid ultrasonography has not been broadly employed clinically for the detecting of atherosclerosis in investigations on patients with *H. pylori* [35]. Since the superficial position of the carotid artery is easy to detect and can be easily obtained by colour Doppler measurement, thus, we used the colour Doppler ultrasound examination of cervical blood vessels in the present study. Notably, the level of cervical vascular lesion formation in the *H. pylori*positive group was significantly higher than that in the *H. pylori*negative group. Zhu et al. [9] Vizzardi et al. [36] and others have concluded that *H. pylori* infection is a risk factor for the formation and development of cervical vascular plaque, which supports the conclusion of this study. The possible reasons are as follows: *H. pylori*can directly invade blood vessels to mature monocytes, accelerate the proliferation of vascular smooth muscle cells or endothelial cells, and promote thrombosis [36]; *H. pylori* infection increases blood lipid levels and fibrin levels, and promotes the formation of atherosclerosis [20].

## 5. Conclusion

In this study, we conclude that *H. pylori* infection is an independent risk factor for higher BMI (>25), triglyceride (>1.7 mmol/l), and neck vascular plaque formation. The multi-variant analysis showed that patients with *H. pylori* infection are prone to have higher BMI, triglycerides, and neck vascular plaque formation more than 1.4-times higher in non-infected individuals. Therefore, actively carrying out *H. pylori* infection screening and eradication treatment has important clinical significance and has good application prospects. Thus, *H. pylori* infection not only could be counted as an independent risk factor for vascular plaque formation, but also it could accelerate vascular plaque formation through increasing BMI and triglyceride.

## Data Availability

The data based on the results of the current study were obtained, are accessible from the corresponding authors upon reasonable request.
